# Investigation the EMG Activities of Lower Limb Muscles When Doing Squatting Exercise in Water and on Land

**DOI:** 10.3390/ijerph16224562

**Published:** 2019-11-18

**Authors:** Calvin H.N. Yuen, Christine P.Y. Lam, Kate C.T. Tong, Jessica C.Y. Yeung, Chloe H.Y. Yip, Billy C.L. So

**Affiliations:** Department of Rehabilitation Sciences, The Hong Kong Polytechnic University, Hong Kong, China

**Keywords:** aquatic exercise, closed-kinetic chain, knee exercise, motion analysis, muscle activity

## Abstract

(1) *Background*: Squatting is one of the common closed-kinetic chain (CKC) exercises for knee rehabilitation. Some patients cannot perform squatting exercises on land occasionally due to knee pain. Several studies had suggested that lower limb muscle activities are lower in water than on land while performing CKC exercises. The purpose of this study is to investigate the surface electromyography (sEMG) activities of Rectus femoris (RF) and Biceps femoris (BF) muscles when doing a squatting exercise in water and on land. (2) *Methods*: This was a cross-sectional experimental study. A total of 20 healthy participants (10 males, 10 females) were recruited by convenience sampling. The sEMG of RF and BF muscles in water and on land were collected and the knee motions were videotaped. Participants were instructed to perform closed kinetic-chain back squatting exercises at a specific speed (30 beats per minute) in water and on land at angular speed of 45°/s. Eight repetitions of the squatting exercise (0–90° knee flexion) were performed. The mean percentage maximal voluntary contraction (%MVC) between two muscles was compared in two conditions. The %MVC of RF and BF muscles at different specific knee flexion angles (30°, 60° and 90° knee flexion) was also identified. (3) *Result*: Muscle activities of RF (*p* = 0.01) and BF (*p* < 0.01) muscles were significantly lower in water than on land. The %MVC of RF and BF muscles was found to be 15.01% and 10.68% lower in water than on land respectively. For different knee angle phases, the differences in %MVC between land and water had significant difference for both RF muscles and BF muscles. (4) *Conclusion*: This study found a difference of mean percentage MVC of RF and BF muscles between land and water in different phases of squatting. The water medium reduced the two muscles’ activities to a similar extent. The result showed that the aquatic environment allows an individual to perform squatting with less muscle activation which may serve as an alternative knee exercise option for patients who encounter difficulty in land squatting due to lower limb muscle weakness or a high level of knee pain.

## 1. Introduction

Aquatic exercises are recommended during the initial phase of a musculoskeletal rehabilitation program, as the specific properties of water allow early exercises for patients who are unable to exercise successfully on land [1,2,3,4,5]. Several studies have suggested the unique physical properties of water, including buoyancy, hydrostatic pressure and thermodynamics, could induce different physiological and biomechanical responses of the body during exercises [6,7,8]. For example, the drag force in water, which is affected by viscosity and buoyancy, could be utilized to alter the resistance of underwater actions and influences muscle activities to produce targeted effects in rehabilitation [6,9]

Many studies have investigated and compared the muscle activities of different muscle groups when performing different types of exercises in water to that on land [2,10,11,12,13]. For example, Cuesta-Vargas and Cano-Herrera [11] investigated muscle activities of trunk muscles when performing deep water running by wearing a buoyancy device to prevent the feet from touching the floor of the pool, walking in water and on land. However, relatively limited studies have investigated the lower limb muscles’ activities in water.

Lower limb muscle strengthening exercises are divided into Open Kinetic Chain (OKC) exercises and Closed Kinetic Chain (CKC) exercises. For CKC exercises, the distal part of the limb is fixated by contacting an immobile surface, so only the proximal part of the body can bring about the movement [14]. There are two studies investigating the thigh muscle activities during sit-to-stand CKC exercise [13,15]. These studies showed that when performing sit-to-stand action in water, the muscle activities of the Biceps femoris (BF) and other muscles were significantly lower than those on land. Most evidence tends to reveal that muscle activities are lower in water. It may also be applicable to other CKC exercises. The relatively lower muscle activities in water may be due to buoyancy on the neuromuscular system and reduced weight bearing [16], and electromechanical factors including hydrostatic pressure during water immersion [17].

Squatting, which is a common lower-limb CKC exercises, can activate the co-contraction of Rectus femoris (RF) and Biceps femoris (BF) muscles. Several land-based studies showed that CKC exercises were effective in achieving better BF activation during the co-contraction of RF and BF muscles, which is essential for athletes to achieve in order to prevent knee injury [18,19]. Squatting exercises may also enhance one’s knee stability [20]. Therefore, squatting exercises are usually incorporated in knee rehabilitation programs [21].

While there is limited research looking into CKC knee exercises in water, and squatting can be an effective exercise for training, it is worth investigating the muscle activities when performing squatting in water as compared to that on land. Hence, we aimed to investigate the surface electromyography (sEMG) activity and the difference of the percentage of maximal voluntary contraction (%MVC) between RF and BF when squatting in water and on land. We also aimed to compare the muscle activity at different knee angles during the squatting activities. We hypothesized that during squatting, muscle activities of RF and BF are lower in water than that on land.

## 2. Materials and Methods

### 2.1. Study Design

This was a cross-sectional study design following the guideline of the Strengthening the Reporting of Observational studies in Epidemiology (STROBE) [22].

### 2.2. Sample Size Planning

Referring to the study of Cuesta-Vargas et al. [13], which investigated sit-to-stand action in water, the mean and standard deviation of %MVC of RF were 13.2+/−5.5 (land) & 4.4+/−1.2 (water) with Cohen’s (d) effect size 1.15; while that of BF were 5.37+/−2.5 (land) & 9.39+/−0.42 (water) with Cohen’s (d) effect size 2.40. By using G*Power (Version 3.1), a total of at least 15 participants were required to detect significant difference in muscle activities of RF and BF in water and on land during CKC exercises at a power of 0.8 and alpha level of 0.05.

### 2.3. Participants

Twenty healthy participants (10 males, 10 females with mean age: 21.25 ± 1.0 year; height: 168.1 ± 6.9 cm; weight: 58.7 ± 7.9 kg) were recruited by convenience sampling based on the following inclusion criteria: (1) aged between 18–40 and (2) able to perform squatting movements in water independently. Candidates with any infectious diseases and skin conditions, any known hip or knee injuries (included previous hip/ knee surgeries) in recent 2 years, or any contraindications to aquatic exercises were excluded from the study. All participants had given informed consent before being enrolled in the study. An information sheet was provided and written informed consent was obtained from recruited providers prior to the start of the trial. The protocol for this study was approved by the Departmental Research Committee of the Hong Kong Polytechnic University’s Department of Rehabilitation Sciences (Reference Number: HSEARS 20170626001).

### 2.4. Experimental Setup

Room and water temperature were maintained at 29.5 °C and 33.8 °C respectively. Participants were asked to perform squatting movements on land, followed by maximal voluntary contractions (MVC) of RF and BF muscles on land, and finally performed squatting in water. The squatting actions were videotaped using waterproofed camera GoProHERO3 at 90 frames/s. The camera was placed 2.5 m away from participants and positioned at knee crease level to prevent any angulation of the video. The sEMG activities of RF and BF of the dominant leg during squatting were recorded using a two-channel sEMG system (SX230 surface EMG sensor, Biometrics, UK) and a customized data logger at 1000 Hz sampling rate. The sEMG signals were then exported using LabView8.6 (National Instruments Corporation, Austin, TX, USA).

### 2.5. Procedures

The procedures of the study were explained to the participants by a standardized instructor. Demographic information including age, height, weight, and leg dominance of the participants were obtained.

#### 2.5.1. Electrodes and Markers

**Figure 1:**
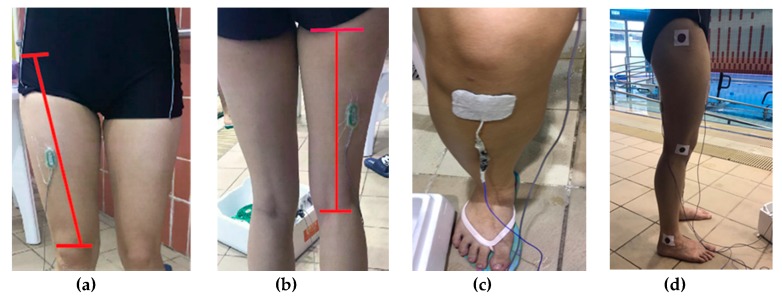
Location of the (**a**) RF electrode placement, (**b**) BF electrode placement, (**c**) ground electrode placement, (**d**) Three bony landmarks with markers attached.

The EMG electrodes were attached over RF and BF muscles of the dominant leg of participants. The location of the RF’s electrode was at the midway between the anterior superior iliac spine and the upper edge of patella (Figure 1a). The location of the RF’ electrode was at the midway between gluteal fold and knee crease (Figure 1b). The location of the ground electrode was over tibial tuberosity (Figure 1c). The required skin areas were shaved, handled with abrasive material (3M Red Dot Trace Prep) and cleaned with alcohol swab (70%isopropyl). For the bony landmarks of (1) greater trochanter of femur, (2) lateral epicondyle of femur and (3) lateral malleolus, markers of 3cm in diameter were attached (Figure 1d) and covered with tegaderm (Smith and Nephew Flexifix Opsite Transparent Adhesive Film Roll 4” x10.9 Yard, model66000041) for kinematic tracking.

#### 2.5.2. Waterproof Techniques

**Figure 2:**
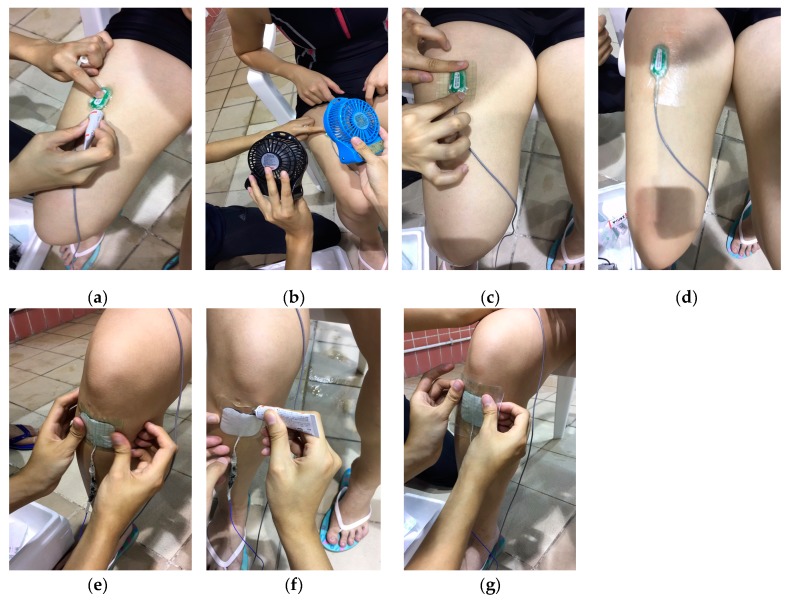
(**a**) Application of the liquid bandage around the electrode. (**b**) Dry up process of liquid bandage. (**c**) Application of the first layer of tegaderm (55 mm × 60 mm) on electrode. (**d**) Application of the second layer of tegaderm after application of liquid bandage along the edges of the first layer of tegaderm. (**e**) Application of the first layer of tegaderm (65 mm × 80 mm) on ground electrode. (**f**) Application of liquid bandage along the edges of tegaderm on ground electrode. (**g**) Application of the second layer of tegaderm (85 mm × 90 mm) on ground electrode.

In reference to previous studies [10,23], the waterproof technique was adopted and modified in order to record sEMG signals under water. Details are shown in Figure 2.

#### 2.5.3. Squatting on Land

Back squat is a squatting strategy which requires the participants to lean the trunk forward while performing squatting. It was selected as the standardized squatting strategy in this study due to balancing issues in water.

Standardized instructions on the posture and speed of performing required movements on land were given to participants. The participants were instructed to stand at shoulder width with arms crossed on their chest. The squatting rhythm was set at 30 beats/min (bpm) using a metronome with video cues so that the participants squatted at an angular speed of 45°/s [24]. Several practicing trials were given to participants to familiarize themselves with the movements. Eight squats (0–90° knee flexion) were performed and videotaped. sEMG activities of RF and BF were also recorded. A 10-min rest was given to participants before taking measurements in water.

#### 2.5.4. Squatting in Water

Standardized instructions on the posture and speed of performing the required movement in water, which are same as those on land, were given to participants. The water level should be at umbilical level when standing upright. Participants were required to stand on either a 15-cm or a 25-cm tall platform if the water level was too high. Several practicing trials were given to participants to familiarize themselves with the aquatic pool and the movements. Eight squats were performed and videotaped. sEMG activities of RF and BF were also recorded.

#### 2.5.5. Maximal Voluntary Contractions (MVC)

Participants were asked to perform three 5-s MVC of RF and BF separately on land to normalize sEMG data recorded during squatting on land and in water. A 2-min rest was given between each MVC. Procedures of MVCs of RF and BF are listed as follows:

##### RF MVC Testing

**Figure 3:**
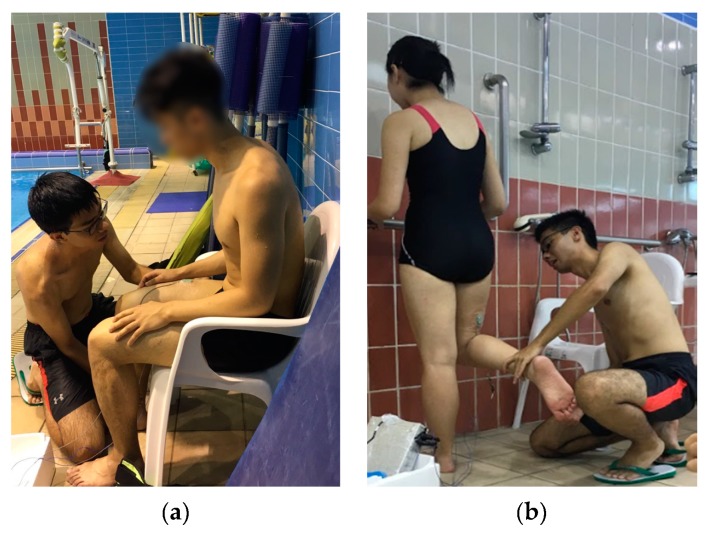
(**a**) RF MVC testing. (**b**) BF MVC testing.

Participants were instructed to sit on a chair with hips and knees flexed at 90° and the trunk supported by the chair back. Arms were on thighs to prevent substituted movements. A standardized investigator manually resisted isometric knee extension for 5 s at self-reported maximum exertion by participants (Figure 3a).

##### BF MVC Testing

Participants stood on the non-dominant leg and with the dominant knee flexed at 90°. Participants were allowed to support themselves against a handrail on a wall using their arms for balance. The investigator manually resisted knee flexion isometrically for 5 s after participants informed that maximum exertion was reached (Figure 3b).

According to Silver and Dolny [4], no significant difference was found in the results of MVC of RF and BF on land and in water. Therefore, we used the land MVC values to normalize muscle activation data recorded during the squatting exercise performed in water and on land.

### 2.6. Data Processing

Raw sEMG signals were processed by bandpass filter (at 20 to 300 Hz) and root-mean-square sliding window (50 ms time constant) (MatLab2017a; Mathematical computing software, Natick, MA, USA). A customized program was used to determine the period of the middle four squats. Amplitudes of EMG signal for the RF and BF were calculated and averaged. Raw MVC data were filtered and smoothed in the same way as raw sEMG signals. MVC values of the three bursts of contractions were first calculated into three separate means. The greatest mean MVC value among the three bursts was selected as the MVC value of the RF and BF. Mean sEMG amplitudes for the four squats were normalized to these MVC values and expressed as %MVC. For kinematic data, knee angles at the corresponding time were analyzed from markers on participants in the videos taken using motion-tracking software Kinovea (v.0.8.25) (Kinovea, Bordeaux, Nouvelle Aquitaine, France). The data were synchronized with sEMG data for statistical analysis.

### 2.7. Statistical Analysis

All statistical calculations were computed using Matlab 2017a (Mathematical computing software, Natick, MA, USA). Descriptive data were calculated for demographic data (age, height and weight) and %MVC. The data were presented in the format of mean ± standard deviation.

Total mean of sEMG activities of RF and BF muscles in terms of %MVC were analyzed to compare between two squatting environments, which were land and water, using paired t-test for parametric data and Wilcoxon test for non-parametric data.

The distribution patterns of the %MVC of RF and BF muscles of ascending and descending phases and different specific knee flexion angles (30° and 60° of knee flexion during ascending and descending phase and 90° knee flexion) during the squatting exercises were identified and confirmed with Matlab 2017a. Then, a generalized linear model was applied to compare (1) %MVC of RF and BF muscles between the ascending and descending phase on land and in water and (2) %MVC of RF and BF muscles among different knee flexion angles and exercise environments (land and water). Alpha level was set at 0.05 for all statistical computation.

## 3. Results

Table 1 showed the descriptive characteristics of the participants. The mean age of participants is 21.25+/−1.0 years. The mean body height and weight are 168.1+/−6.9 cm and 58.7+/−7.9 kg respectively.

Mean, standard deviation and the difference (as compared to land) of %MVC of RF and BF muscles of the participants at different squatting phases and knee flexion angles at different environment are presented in Table 2. Shapiro-Wilk tests indicated that some data sets did not have parametric distribution. For those data sets requiring a generalized linear model as a comparison method, gamma distribution was applied to fit into the analysis. From Table 2, it was observed that the %MVC of both muscles of the participants were higher on land than in water. When comparing the percentage differences of %MVC in different phases of the squatting exercise and knee flexion angle between land and water, most comparisons show a 5.07% to 36.20% decrease of %MVC of both muscles in water.

Table 3 compared the mean difference of %MVC of RF muscle of the participants at total squatting action in different squatting environments. The total mean of %MVC of RF muscles in water was significantly lower than that on land (*p* = 0.01) either in upward or downward movement phases. The same situation applied to BF, the total mean of %MVC in water was significantly lower than on land (*p* < 0.01) (Table 4).

**Figure 4:**
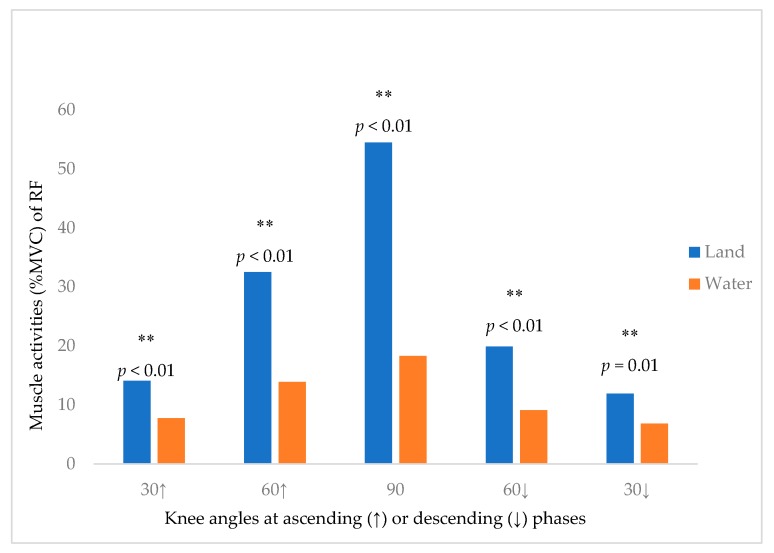
The Muscle Activity of RF (%MVC) at different knee angles (degree) during squatting. ** significant at *p* < 0.01; (↑) ascending phase; (↓) descending phase.

**Figure 5:**
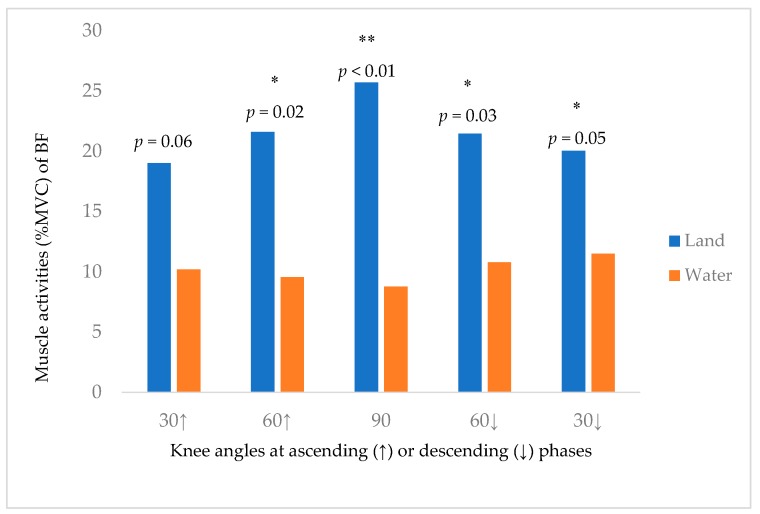
The Muscle Activity of BF (%MVC) at different knee angles (degree) during squatting. * significant at *p* < 0.05, ** significant at *p*<0.01; (↑) ascending phase; (↓) descending phase.

Figure 4 showed the differences of RF muscle activity between land and water environments at different knee angles (30° upward phase, 60° upward phase, 90°, 60° upward phase, 30° downward phase). There is a significantly lower %MVC of RF (5.07–36.20%) in water environment. Figure 5 showed the differences of BF muscle activity between land and water environments at different knee angles (30° upward phase, 60° upward phase, 90°, 60° upward phase, 30° downward phase). There is a significantly lower %MVC of BF (8.54–16.93%) in water environment.

## 4. Discussion

### 4.1. Difference of RF and BF Activity in Water and on Land

The findings of this study showed that squatting environments affect the activities of RF and BF muscles in overall action. Lower muscle activities were found when squatting in water as compared to squatting on land. This finding was coherent with another study investigating the leg muscle activities during sit-to-stand movement in water [13]. In our study, the result showed that both RF and BF co-contracted during the whole process of squatting either in water or on land.

### 4.2. RF and BF Activity in Water and on Land at Different Squatting Phases and Knee Angles

The muscle activity of RF and BF were higher with the increase in knee flexion angle particularly highest at 90° knee flexion position in both water and land environments. However, the muscle activity of BF in water remained at around 10%MVC throughout the whole process of squatting. In land squatting, the BF muscle co-contracted (around 20–25%MVC) with RF against gravity and maintain knee position; however, the constant and relatively lower level of BF muscle activity may be due to the drag force generated due to knee movement. BF acted as a stabilizer during the water squatting to overcome the potential drag force. In this study, we further analyzed the reduction rate of muscle activity in water environment at different knee flexion angles when comparing with land environment. The result showed that there was the largest reduction of muscle activity of RF (36.20%) and BF (16.93%) at 90° knee flexion position. The reduction of muscle activity may be due to the change of knee angle and also due to different water properties of water environment [7].

### 4.3. The Effect of Different Water Properties on the RF and BF Muscle Activity

Muscle activities in water are affected by different water properties. One possible reason for lower muscle activities in water is the effect of drag force in term of water resistance. Drag force is the force on an object that resists its motion through a fluid. The drag force is positively proportional to the movement speed [6]. Higher movement velocity would produce higher drag force in water, thus creating larger resistance for that movement, and vice versa [6]. In our study, the participants completed one squat in 4 s. It is possible that the squatting velocity was relatively low, resulting in lower drag force and thus less resistance to the squatting actions. Other water properties, such as buoyancy, also altered the movement resistance so the drag force might not be high enough to create enough resistance to increase the muscle activities. Further studies could investigate the sEMG activities of the RF and BF muscles in water with different squatting speeds.

Buoyancy of water is the upward force of water that opposes the weight of the immersed object [25]. In the ascending phase of a squat (squatting up), RF muscles are expected to work less hard in water than on land as this muscle action is assisted by buoyancy. In our study, although no significant difference was shown for RF muscle activities between the ascending phase and descending phase in water, a trend of smaller RF muscle activities was shown in the ascending phase (%MVC = 10.20 ± 6.08) than descending phase (%MVC = 12.87 ± 8.28) in water.

Other studies also support that buoyancy affects muscle activities by reducing body weight [17,26]. Weight reduction exerts an effect on muscle spindles in the neuromuscular system in which the muscle spindle activities and stimulation to receptors within muscles could be reduced [26]. Reduced weight-bearing condition results in a reduction of stimulation to pressure receptors in skin and gravito-receptors in muscles and the vestibular system, thereby triggering an inhibitory mechanism and lowering the reflex and proprioceptive mechanism. This chain of mechanism finally leads to reduced muscle activities [26]. In our study, participants were submerged at the water level of umbilicus level. The body weight reduction was approximately 50% [8]. This reduction of body weight might lead to lower BF activities in water. Future studies could investigate the relationship between different immersion depths and muscle activities.

In clinical application, knowing that doing a squatting exercise in water could reduce lower limb muscle activities may help clinicians or therapists in designing a rehabilitation program for people with knee injuries presenting with a high pain score or lower limb weakness, especially in the early phase of the injury or post-operative period. Further research on the correlation in pain score of patients with knee pain while performing exercises in water with that on land could be done to provide more clinical evidence. In addition, by acknowledging that squatting speed and immersion depth could alter the extent of reduction in muscle activities, further research on this field could help in controlling these parameters in designing an optimal rehabilitation program for people with knee problems. Future EMG studies on other lower limb muscles should be considered for comparing squatting or other functional activity (e.g. gait pattern) in both environments [27,28].

### 4.4. Limitation of Study

There were several limitations in our study. Regarding the squatting strategies, individual variance on squatting methods may have existed. Although we standardized the squatting method as “back squat”, some participants may use their own squatting strategies while performing the squatting movement in water. In addition, owing to the limitation of equipment, the interelectrode distance of the electrodes was fixed at 2 cm. Hence, electrode placement on RF muscles failed to follow the guidelines of Criswell [29], who suggests a 10–15 cm interelectrode distance for recording RF muscles in general. With the 2-cm interelectrode distance at the location of our RF muscles’ electrode placement, the sEMG recorded would likely be the RF muscle instead of the RF muscle group. The results of our study showed lower muscle activities in water in RF and BF muscles at a squatting speed of 4 s/squat. Our findings, therefore, cannot be generalized to subjects using different squatting methods, different squatting speeds, performing other actions, and different immersion depths. Besides, regarding the measurement method, this study did not employ ultrasound to monitor muscle quality and quantity, which has now widely been used for muscle measurement [30].

## 5. Conclusions

This study found lower RF and BF muscle activities of healthy individuals when performing squatting in water than on land. This study also found the percentage difference of RF and BF muscle activity between land and water in different phases (descending and ascending) of squatting. Overall, a 5.07% to 36.20% decrease in %MVC was found in water for both muscles. The water medium affects the two muscles’ activities to a similar extent. Further investigation about other factors affecting the %MVC of these two muscles are needed, including the methods of squatting, the water immersion depth, the speed of squatting etc. Overall, an aquatic closed-chain knee exercise provides an alternative to knee rehabilitation on land. Hydrotherapy is recommended as an alternative of exercises for some patients who are at the initial phase of rehabilitation, including acute post-surgery stage (e.g., ACL reconstruction [5]). For patients who are too weak to initiate lower limb close-chain rehabilitation exercises on land, aquatic exercises might allow early close-chain rehabilitation to facilitate faster functional return. Lower muscle activities can also be safer and less demanding, which is more suitable for some groups of patients who cannot tolerate high muscle activities or land exercises.

## Figures and Tables

**Table 1:** Descriptive characteristics of the 20 participants.

Demographic Factors	Minimum	Maximum	Mean	Std. Deviation
Age (year)	20	24	21.25	1.0
Height (cm)	150	178	168.1	6.9
Weight (kg)	46	70	58.7	7.9
Body mass index (kg/m^2^)	17.5	24.6	20.7	2.0

Std. deviation (Standard deviation).

**Table 2:** Mean, standard deviation and the percentage difference (as compared to land) of %MVC of RF and BF muscles at different phases in water compared to land.

Phase/Muscles	Land (%MVC, Mean ± SD)		Water (%MVC, Mean ± SD)		% MVC Differences in Water Compared to Land (Mean ± SD)	
	RF	BF	RF	BF	RF	BF
Total phase	26.45 ± 13.51	20.62 ± 13.79	11.44 ± 6.12	9.97 ± 13.93	15.01 ± 10.47	10.68 ± 16.64
↑phase	21.45 ± 11.87	19.93 ± 11.87	10.20 ± 6.08	10.60 ± 14.12	11.25 ± 5.79	9.33 ± 15.46
↓phase	28.88 ± 18.29	19.38 ± 12.69	12.87 ± 8.28	9.67 ± 14.07	16.01 ± 10.01	9.71 ± 15.64
30° ↓	14.07 ± 6.76	19.02 ± 15.83	7.76 ± 4.17	10.20 ± 13.20	6.31 ± 6.53	8.82 ± 17.09
60° ↓	32.54 ± 19.83	21.60 ± 16.11	13.86 ± 10.54	9.55 ± 13.76	18.68 ± 20.79	12.06 ± 18.28
90°	54.5 ± 31.09	25.70 ± 18.01	18.29 ± 11.08	8.77 ± 14.28	36.20 ± 26.12	16.93 ± 20.56
60° ↑	19.90 ± 12.61	21.46 ± 14.30	9.12 ± 8.06	10.78 ± 14.65	10.78 ± 12.69	10.68 ± 17.33
30° ↑	11.88 ± 7.46	20.04 ± 12.87	6.81 ± 4.68	11.50 ± 13.87	5.07 ± 6.38	8.54 ± 15.67

(↑) ascending phase; (↓) descending phase. MVC: Maximal Voluntary Contraction.

**Table 3:** The comparison of RF muscle activity at different media at different squatting phases.

Phase/Muscles	RF (%MVC, Mean ± SD)		Difference in % MVC of RF at Two Media (Mean ± SD, *p*-Value)	
	Land	Water		
Total phase	26.45 ± 13.51	11.44 ± 6.12	15.01 ± 10.47	0.01 **
↑phase	21.45 ± 11.87	10.20 ± 6.08	11.25 ± 5.79	<0.01 **
↓phase	28.88 ± 18.29	12.87 ± 8.28	16.01 ± 10.01	<0.01 **

** significant at *p* < 0.01; (↑) ascending phase; (↓) descending phase. RF: Rectus Femoris.

**Table 4:** The comparison of BF muscle activity at different media at different squatting phases.

Phase/Muscles	BF (%MVC, Mean ± SD)		Difference in % MVC of BF at Two Media (Mean ± SD, *p*-Value)	
	Land	Water		
Total phase	20.62 ± 13.79	9.97 ± 13.93	10.68 ± 16.64	0.01 **
↑phase	19.93 ± 11.87	10.60 ± 14.12	9.33 ± 15.46	<0.01 **
↓phase	19.38 ± 12.69	9.67 ± 14.07	9.71 ± 15.64	<0.01 **

** significant at *p* < 0.01; (↑) ascending phase; (↓) descending phase. BF: Biceps Femoris.

## References

[1] Heywood S., McClelland J., Geigle P., Rahmann A., Villalta E., Mentiplay B., Clark R. (2019). Force during functional exercises on land and in water in older adults with and without knee osteoarthritis: Implications for rehabilitation. Knee.

[2] Heywood S., McClelland J., Mentiplay B., Geigle P., Rahmann A., Clark R. (2017). Effectiveness of Aquatic Exercise in Improving Lower Limb Strength in Musculoskeletal Conditions: A Systematic Review and Meta-Analysis. Arch. Phys. Med. Rehabil..

[3] Batterham S.I., Heywood S., Keating J.L. (2011). Systematic review and meta-analysis comparing land and aquatic exercise for people with hip or knee arthritis on function, mobility and other health outcomes. BMC Musculoskelet. Disord..

[4] Silva L.E., Valim V., Pessanha A.P.C., Oliveira L.M., Myamoto S., Jones A., Natour J. (2008). Hydrotherapy versus conventional land-based exercise for the management of patients with osteoarthritis of the knee: A randomized clinical trial. Phys. Ther..

[5] Momberg B.L., Louw C., Crous L. (2008). Accelerated hydrotherapy and land-based rehabilitation in soccer players after anterior cruciate ligament reconstruction: A series of three single subject case studies. South Afr. J. Sports. Med..

[6] Alberton C.L., Cadore E.L., Pinto S.S., Tartaruga M.P., Da Silva E.M., Kruel L.F.M. (2011). Cardiorespiratory, neuromuscular and kinematic responses to stationary running performed in water and on dry land. Eur. J. Appl. Physiol..

[7] Becker B.E. (2009). Aquatic therapy: Scientific foundations and clinical rehabilitation applications. PM&R.

[8] Harrison R., Bulstrode S. (2009). Percentage Weight-Bearing during Partial Immersion in the Hydrotherapy Pool. Phys. Pract..

[9] Heywood S., McClelland J., Geigle P., Rahmann A., Clark R. (2016). Spatiotemporal, kinematic, force and muscle activation outcomes during gait and functional exercise in water compared to on land: A systematic review. Gait Posture.

[10] So B.C.L., Yuen C.H.N., Tung K.L.H., Lam S., Cheng S.L., Hung Z.W.L., Leung R.W.K., Szeto G.P.Y. (2019). A Study on Trunk Muscle Activation of 2 Deep Water Running Styles (High-Knee and Cross-Country Style) and Land Walking. J. Sport. Rehabil..

[11] Cuesta-Vargas A.I., Cano-Herrera C.L. (2014). Surface electromyography during physical exercise in water: A systematic review. BMC Sports Sci. Med. Rehabil..

[12] Hoskin K., Dodd K., Chan S.P., Rosengarten S., Heywood S. (2013). Aquatic Exercise Compared to Contrast Therapy With Shallow Water Treadmill Running to Assist. Recovery in Elite Australian Rules Footballers. Int. J. Aquat. Res. Educ..

[13] Cuesta-Vargas A.I., Cano-Herrera C.L., Heywood S. (2013). Analysis of the neuromuscular activity during rising from a chair in water and on dry land. J. Electromyogr. Kinesiol..

[14] Lee N.K., Kwon J.W., Son S.M., Kang K.W., Kim K., Hyun-Nam S. (2013). The effects of closed and open kinetic chain exercises on lower limb muscle activity and balance in stroke survivors. NeuroRehabilitation.

[15] Cuesta-Vargas A.I., Cano-Herrera C.L. (2019). EMG Analysis of the Neuromuscular Activity during Sit-to-Stand from Different Height Chairs in Water. Int. J. Aquat. Res. Educ..

[16] Pöyhönen T., Kyröläinen H., Keskinen K.L., Hautala A., Savolainen J., Mälkiä E. (2001). Electromyographic and kinematic analysis of therapeutic knee exercises under water. Clin. Biomech..

[17] Pöyhönen T., Avela J. (2002). Effect of head-out water immersion on neuromuscular function of the plantarflexor muscles. Aviat. Space Environ. Med..

[18] Shields R.K., Madhavan S., Gregg E., Leitch J., Petersen B., Salata S., Wallerich S. (2005). Neuromuscular control of the knee during a resisted single-limb squat exercise. Am. J. Sports Med..

[19] Escamilla R.F., Fleisig G.S., Zheng N., Barrentine S.W., Wilk K.E., Andrews J.R. (1998). Biomechanics of the knee during closed kinetic chain and open kinetic chain exercises. Med. Sci. Sports Exerc..

[20] Gullett J.C., Tillman M.D., Gutierrez G.M., Chow J.W. (2009). A biomechanical comparison of back and front squats in healthy trained individuals. J. Strength Cond. Res..

[21] Hinman R.S., Heywood S.E., Day A.R. (2007). Aquatic physical therapy for hip and knee osteoarthritis: Results of a single-blind randomized controlled trial. Phys. Ther..

[22] Von Elm E., Altman D.G., Egger M., Pocock S., Gøtzsche P.C., Vandenbroucke J.P. (2014). The Strengthening the Reporting of Observational Studies in Epidemiology (STROBE) Statement: Guidelines for reporting observational studies. Int. J. Surg..

[23] Silvers W.M., Dolny D.G. (2011). Comparison and reproducibility of sEMG during manual muscle testing on land and in water. J. Electromyogr. Kinesiol..

[24] Nishiwaki G.A., Urabe Y., Tanaka K. (2006). EMG Analysis of Lower Extremity Muscles in Three Different Squat Exercises. J. Jpn. Phys. Ther. Assoc..

[25] Torres-Ronda L., Del Alcazar X.S. (2014). The Properties of Water and their Applications for Training. J. Hum. Kinet..

[26] Masumoto K., Takasugi S.I., Hotta N., Fujishima K., Iwamoto Y. (2005). Muscle activity and heart rate response during backward walking in water and on dry land. Eur. J. Appl. Physiol..

[27] Roca-Dols A., Losa-Iglesias M.E., Sánchez-Gómez R., Becerro-de-Bengoa-Vallejo R., López-López D., Palomo-López P., Rodríguez-Sanz D., Calvo-Lobo C. (2018). Electromyography activity of triceps surae and tibialis anterior muscles related to various sports shoes. J. Mech. Behav. Biomed. Mater..

[28] Roca-Dols A., Losa-Iglesias M.E., Sánchez-Gómez R., López-López D., Becerro-de-Bengoa-Vallejo R., Calvo-Lobo C. (2018). Electromyography comparison of the effects of various footwear in the activity patterns of the peroneus longus and brevis muscles. J. Mech. Behav. Biomed. Mater..

[29] Criswell E., Cram J.R. (2011). Cram’s Introduction to Surface Electromyography.

[30] Chang K.V., Yang K.C., Wu W.T., Huang K.C., Han D.S. (2019). Association between metabolic syndrome and limb muscle quantity and quality in older adults: A pilot ultrasound study. Diabetes Metab. Syndr. Obes..

